# Integrative transcriptomic and functional analyses identify *TaARF* in the auxin response pathway as a hub gene conferring multi-stage heat tolerance in wheat

**DOI:** 10.3389/fpls.2026.1965437

**Published:** 2026-10-13

**Authors:** Zhengxian Xu, Yunfeng Qu, Miao He, Min Fan, Jinghuan Zhu, Weidong Wang, Xin Hu, Delin Li, Qiuhong Wu, Hongjie Li, Yongxing Chen, Junkang Rong, Wei Hua

**Affiliations:** 1 College of Advanced Agricultural Sciences, Zhejiang Agriculture and Forestry University, Lin’an, China; 2 Institute of Crop and Nuclear Technology Utilization, Zhejiang Academy of Agricultural Sciences, Hangzhou, China; 3 Institute of Biotechnology, Xianghu Laboratory, Hangzhou, Zhejiang, China

**Keywords:** auxin response, developmental stages, heat tolerance, transcriptomics, *Triticum aestivum* L., WGCNA

## Abstract

Heat stress severely impacts the growth, development, and yield of wheat (*Triticum aestivum* L.) throughout its lifecycle. Identifying key genes regulating heat tolerance is crucial for breeding climate-resilient wheat cultivars. In this study, Zhenongmai 1 (ZNM1), a heat-tolerant wheat cultivar from Zhejiang Province, exhibited a significantly higher seedling survival rate than the heat-sensitive Yangmai 20 after heat stress. At the maturity stage, ZNM1 maintained superior grain yield components under heat stress, retaining 90.05% of its thousand-grain weight (TGW), while YM20 only retained 84.53%. To elucidate the molecular regulatory mechanism underlying heat tolerance in ZNM1, Weighted Gene Co-expression Network Analysis (WGCNA), Gene Ontology (GO), and Kyoto Encyclopedia of Genes and Genomes (KEGG) enrichment analyses, were performed to screen heat stress-responsive candidate genes. The results revealed that these candidate genes were predominantly enriched in biological processes including auxin metabolism and NAD(P)H dehydrogenase complex assembly, which are critical for wheat heat tolerance regulation. The expression patterns of 11 core candidate genes were verified by quantitative real-time PCR (RT-qPCR), validating the credibility of the transcriptomic data. Heterologous yeast expression assays further confirmed that *TaARF* positively regulated plant heat tolerance. Additionally, we systematically analyzed the haplotype variations of *TaARF* among wheat germplasms from different geographical environments and characterized its tissue-specific expression profiles. This study clarifies the key genes and potential molecular pathways associated with wheat heat tolerance, providing valuable gene resources and theoretical references for the genetic improvement of heat-resistant wheat varieties.

## 1 Introduction

Wheat (*Triticum aestivum* L.) is one of the most important staple cereal crops worldwide, contributing approximately 20% of global caloric intake and serving as a primary source of nutrition and economic stability. Sustained wheat production is therefore fundamental to global food security, particularly in the context of a rapidly growing population (Matros et al., 2017; Pagano et al., 2026; Al-Huqail et al., 2023). However, climate change has led to an increased frequency and intensity of extreme heat events, posing a serious threat to wheat productivity (Asseng et al., 2019; Lobell et al., 2011; Abeed et al., 2020). Elevated temperatures—especially during flowering and grain-filling stages—disrupt key physiological processes, including photosynthesis, transpiration, assimilate transport, and grain development (Girousse, 2023; Lal et al., 2022; Pradhan and Prasad, 2015). Furthermore, heat stress accelerates water loss through evapotranspiration, exacerbating drought stress and further reducing yield potential (Lepekhov, 2022; Abbas et al., 2023). These interconnected challenges underscore the urgent need to develop heat-resilient wheat cultivars, with genetic improvement recognized as a sustainable and effective strategy.

Substantial progress has been made in elucidating the molecular mechanisms underlying heat tolerance in wheat. Core components of the heat stress response include heat shock proteins (e.g., HSP90) (Hahn et al., 2011), antioxidant enzymes (e.g., TaSOD) (Wang et al., 2022), and key transcription factors such as TaHsfA2h and TaWRKY74-B (Wei et al., 2025; Fu et al., 2024). More recently, complex regulatory networks have been revealed. For example, the kinase TaSG-D1 enhances thermotolerance by phosphorylating and stabilizing the transcription factor TaPIF4 (Zhou et al., 2025). The histone acetyltransferase TaHAG1 activates photosynthesis-related genes (*TaG1* and *TaPSBR1*) via chromatin remodeling, leading to a 12–15% increase in grain weight under heat stress. In addition, the NAC transcription factor *TaNAC18* maintains reactive oxygen species (ROS) homeostasis by promoting the assembly of the NAD(P)H dehydrogenase complex, thereby mitigating mitochondrial oxidative damage (Liu et al., 2025; Al-Huqail et al., 2025).

Despite these insights, the stage-specificity of gene function remains a critical knowledge gap. For instance, *TaHAG1* functions during grain filling, whereas *TaWRKY74-B* is key to root thermotolerance at the seedling stage (Gudi et al., 2025). This context-dependency indicates the need to identify genes and regulatory networks that confer resilience across vegetative and reproductive stages for lifelong heat tolerance. Heat tolerance is a complex trait that involves both constitutive (developmental-stage independent) and inducible (stage-specific) mechanisms. Genes that are constitutively expressed across developmental stages or that participate in conserved stress-signaling pathways (e.g., hormone signaling, ROS homeostasis, and protein protection systems) are likely to function in both vegetative and reproductive tissues (Hussain et al., 2024); Therefore, transcriptomic profiling at the seedling stage can serve as an efficient proxy for identifying conserved regulatory genes that may also contribute to heat tolerance during grain filling. This approach is particularly valuable for initial screening, as seedling-stage assays are faster, more reproducible, and less environmentally confounded than field-based reproductive-stage evaluations. To address this challenge, the seedling stage has become essential for capturing dynamic gene regulatory networks and identifying conserved molecular responses to stress. The advent of high-throughput sequencing technologies has revolutionized functional genomics, enabling comprehensive investigations into the molecular basis of complex traits. RNA sequencing (RNA-seq), in particular, provides a powerful platform for profiling gene expression, uncovering differentially expressed genes (DEGs), and revealing functional pathways involved in stress adaptation (Fowler and Thomashow, 2002; Kreps et al., 2002; Shi et al., 2017; Su et al., 2019).

Weighted Gene Co-expression Network Analysis (WGCNA) further enhances transcriptomic studies by identifying modules of co-expressed genes and linking them to specific phenotypic traits. This approach facilitates discovery of hub genes and regulatory networks underlying plant growth, development, and stress responses (Zong et al., 2023; Liu et al., 2021; Tian et al., 2020; Tahmasebi et al., 2019). It is becoming more and more widely used in plant research applications: in order to know the different co-expression modules of rice under salt stress, Zhu et al. analyzed 457 core DEGs by using WGCNA and successfully obtained three modules, concluding that the resultant three modules were positively correlated with rice salt stress, suggesting that the genes in the modules positively regulated the salt tolerance of rice (Zhu et al., 2019). By analyzing the transcriptomes of Cd-treated different maize varieties for WGCNA analysis, Li et al. divided them into 37 different gene network modules and identified five candidate genes in maize kernels that respond to cadmium stress, which provides a useful dataset for the intrinsic mechanism of cadmium accumulation in maize (Li et al., 2022; Mao et al., 2025). The ARF transcription factor is a core component of the auxin signaling pathway. In recent years, numerous studies have shown that it plays a potential role in regulating plant tolerance to abiotic stress. At the same time, analyzing the haplotype variations of key genes is helpful in revealing the genetic diversity and adaptive evolution characteristics of these genes in germplasm resources.

In this study, firstly, we conducted phenotypic identification of ZNM1 and YM20 during the seedling stage after heat stress, and also statistically analyzed their agronomic traits during the grain-filling stage (such as thousand-grain weight, grain length, and grain width). The results showed that both the survival rate during the seedling stage and the agronomic traits during the grain-filling stage, ZNM1 was more heat-tolerant than YM20. Then, we used WGCNA and enrichment analysis to identify the related modules and candidate genes of ZNM1’s response to high-temperature stress during the seedling stage. These genes were significantly associated with key biological processes (including auxin metabolism and NAD(P)H reductase complex assembly), indicating that they play a role in regulating heat tolerance. The expression patterns of the selected key candidate genes were verified by quantitative real-time polymerase chain reaction (RT-qPCR) and variant calling, and the candidate genes were transferred into yeast using vectors for external expression verification, thereby confirming the reliability of the transcriptome data.

## 2 Materials and methods

### 2.1 Plant materials and heat treatment

Following screening, ZNM1 (pedigree: YM18 × ZM261) is a cultivar bred by the Zhejiang Academy of Agricultural Sciences. Its parental line ZM261 has been previously identified as a heat-tolerant germplasm in preliminary screening, while YM18 is a widely adapted cultivar with good agronomic performance. The heat tolerance of ZNM1 is thus inferred to be largely derived from the ZM261 parent, whereas Yangmai 20 (YM20, pedigree: YM9 × YM10) was selected as the heat-sensitive control. Seeds were surface-sterilized with 1% sodium hypochlorite for 15 min, rinsed five times with distilled water, and incubated at 4 °C in darkness for 3 days to break dormancy and promote germination. Uniformly germinated seeds were grown in 96-well hydroponic trays under controlled conditions: 60% relative humidity, 22/22 °C (light/dark), and a 16-h photoperiod for 7 days. The nutrient solution was refreshed every two days. Seven-day-old seedlings were transferred to quarter-strength Hoagland’s solution and exposed to 42 °C for 5 days, followed by a 5-day recovery at 22 °C. The quarter-strength Hoagland solution was replaced every 2 days. Survival rates were recorded based on viability post-recovery (Cao et al., 2024).

To assess heat tolerance during the reproductive stage, ZNM1 and YM20 were cultivated at the Yangdu Experimental Base of the Zhejiang Academy of Agricultural Sciences. At the grain-filling stage (15–20 d after anthesis), plastic greenhouses were erected to simulate chronic high-temperature stress (average 38 °C/28 °C light/dark) for 25 d. Control plants were grown outside the greenhouses under ambient conditions. After harvest, grains were threshed, and thousand-grain weight (TGW), grain length and width were measured to calculate the percentage loss under heat stress relative to controls (Lee et al., 2022).

### 2.2 Data acquisition and transcriptome sequencing

Leaf samples were collected from seedlings at six time points (0, 0.5, 3, 24, 72, and 120 h) after heat treatment with three biological replicates per sample. The present study contained 36 samples (two cultivars were sampled at six time points, with three replicates each). Total RNA was extracted, and libraries were constructed and sequenced on the DNBSEQ-T7 platform (Novogene, Tianjin, China) to generate paired-end reads. Raw reads were processed using Trimmomatic v0.39 (Bolger et al., 2014) with the following quality filtering criteria: reads were trimmed when the average quality score within a sliding window of 4 bp fell below Q20, and any read shorter than 50 bp after trimming was discarded. Clean reads were aligned to the Chinese Spring reference genome (IWGSC RefSeq v2.1) using HISAT2 (v2.2.1) (Sirén et al., 2014). Transcript abundance was quantified as transcripts per million (TPM) using featureCounts v2.0.6 (Liao et al., 2014). Differential gene expression analysis was performed with DESeq2 (v1.40.2) (Love et al., 2014), applying Benjamini–Hochberg correction for multiple testing. Genes with |log_2_Fold Change| ≥ 1 and false discovery rate (FDR) < 0.05 were considered significantly differentially expressed genes (DEGs).

### 2.3 Construction of weighted gene co-expression network

Gene co-expression networks were constructed using the WGCNA package (v1.6.1) in R (Langfelder and Horvath, 2008). Expression matrices from all samples were used as input. A soft-thresholding power of β = 9 was selected based on scale-free topology criteria (R^2^ > 0.8). Topological overlap matrices were calculated, and hierarchical clustering with dynamic tree-cutting identified gene modules. Highly similar modules were merged (height = 0.25, minimum module size = 30), resulting in 20 distinct co-expression modules. Module-trait associations were determined by correlating module eigengenes with phenotypic traits. Modules with |r| > 0.5 and P < 0.05 were considered significantly correlated with heat tolerance. Visualization of gene interaction networks within key modules was performed using Cytoscape v3.9.0.

### 2.4 Functional annotation and enrichment analysis of modular genes

The correlation coefficients (*r*-values) and corresponding *P*-values between module eigengenes and traits were calculated to determine module specificity. Modules with an |*r*| > 0.5 and *P* < 0.01 were selected as specific modules and analyzed using Gene Ontology (GO) and Kyoto Encyclopedia of Genes and Genomes (KEGG) analyzes. GO and KEGG enrichment analyzes were performed using the ClusterProfile package in the R program (Yu et al., 2010), with multiple testing correction. A corrected *P* < 0.05 was considered significantly enriched.

### 2.5 Real-time fluorescence quantitative PCR validation

To validate RNA-seq results, RT-qPCR was performed on 11 candidate genes exhibiting differential expression between ZNM1 and YM20. Three biological replicates were used per genotype and condition. Reactions were carried out in 20 μL using HeiFiq™ qPCR SYBR Green Master Mix (Yeasen, Shanghai, China) under the conditions: 95 °C for 10 s, followed by 40 cycles of 95 °C for 10 s and 60 °C for 30 s. Gene expression levels were calculated using the 2^−ΔΔCt^ method, with wheat *β-TaActin* gene as the internal control (Livak and Schmittgen, 2001). The β-TaActin primers were as follows: forward, 5’-ACCTTCAGTTGCCCAGCAAT-3’; reverse, 5’-CAGAGTCGAGCACAATACCAGTTG-3’. The same method was applied to compare expression patterns across developmental stages.

### 2.6 Verification of exogenous expression in yeast

The recombinant construct (construction vector) composed of the amplified *TaARFs-1D* gene fragment and the pGADT7 vector was prepared, and it was transferred into the eukaryotic yeast AH109 using the PEG/LiAc method. The strain that only transformed the pGADT7 plasmid was used as the negative control (Nchongboh et al., 2014). The AH109 recombinant cells were pre-cultured in SD/-Leu medium (28 °C, 250 rpm) for 12–14 hours, then the culture was centrifuged, and washed once with 1X phosphate buffer solution (PBS), and then inoculated into the SD/-Leu medium and cultured at 28 °C and 42 °C with a rotation speed of 250 rpm for 12 hours. In addition, 5 μl of each treatment of AH109 yeast cells solution was inoculated at different concentration gradients on the SD/-Leu medium and incubated at 28 °C for 2–3 days.

### 2.7 Genetic haplotype and tissue expression analysis

In order to identify the haplotypes of candidate genes in different regions of the world and their expression patterns in different tissues of wheat, gene haplotype were performed according previous study (Wang et al., 2020). Data were obtained from public database (https://wheat.cau.edu.cn/WheatUnion/?language=en), The gene expression in wheat was downloaded from Wheat Expression Browser (https://www.wheat-expression.com/) and WheatOmics (https://wheatomics.sdau.edu.cn/expression/index.html) database (Ma et al., 2021).

## 3 Results

### 3.1 Phenotypic identification of contrasting heat tolerance across developmental stages

Following varietal pre-screening, ZNM1 and YM20 were identified as heat-tolerant and heat-sensitive genotypes, respectively. Under seedling-stage heat stress, ZNM1 exhibited a markedly higher survival rate (86.21%) compared with YM20 (27.53%) (**Figure 1A**), demonstrating a clear contrast in thermotolerance at the vegetative stage. This differential response persisted during the reproductive stage under terminal heat stress imposed during grain filling (38 °C/28 °C day/night). Grain morphological analysis further revealed pronounced deformation in YM20, with kernel length and width reduced by 8.08% (P = 0.001) and 4.16% (P = 0.03), respectively, compared with unstressed controls. By contrast, ZNM1 maintained near-normal grain morphology, exhibiting only minor reductions of 2.13% in length (P = 0.26) and 3.42% in width (P = 0.24) (**Figure 1B**). Thousand-kernel weight (TGW) declined sharply in YM20, decreasing to 84.53% under heat stress (38.36 ± 1.60 g) relative to the control (45.38 ± 1.94 g; P = 0.0002). In contrast, ZNM1 retained 90.05% of its TGW under heat stress (43.15 ± 1.13 g vs. 47.92 ± 3.97 g in control; P = 0.03) (**Figure 1C**). Collectively, these results demonstrate that ZNM1 confers stable thermotolerance across developmental stages, effectively preserving grain development and yield potential under terminal heat stress.

**Figure 1:**
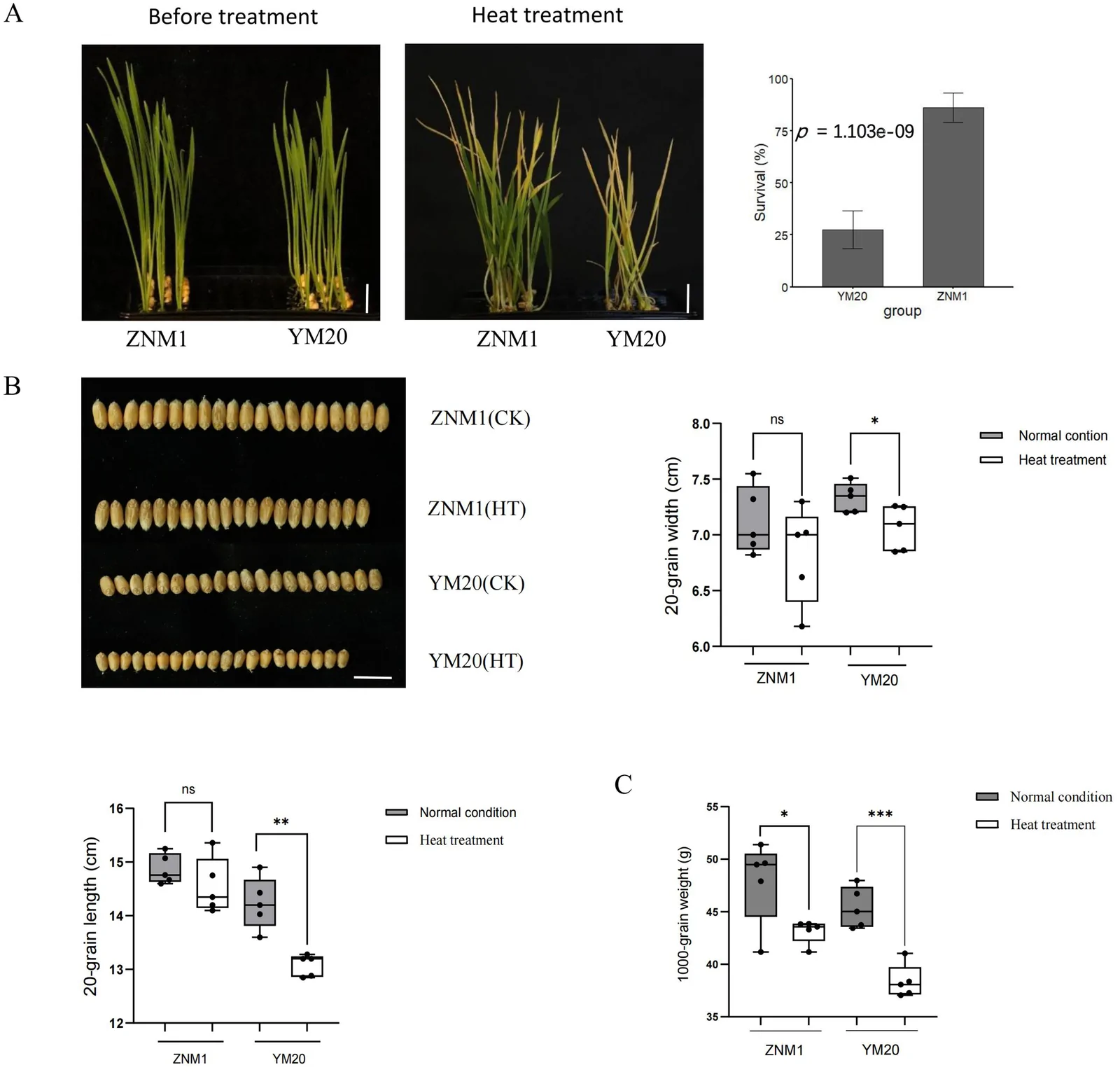
Phenotypes of different wheat varieties after heat stress. **(A)** Survival rate of seedlings under high-temperature stress (42 °C for 5 d). *P* < 0.001. **(B)** Comparison of grain size after heat stress. Twenty grains per group; CK, normal condition; HT, heat stress. Scale bar, 1 cm. **(C)** Statistical analysis of phenotypes and grain weight after field heat stress at the grain-filling stage of ZNM1 and YM20. Analysis was performed using GraphPad Prism 7 (v10.00). Box plots show the interquartile range, including the first quartile, median, and third quartile, with whiskers from minimum to maximum. *P* value indicates the significant yield loss under heat stress between the two cultivars. Statistical significance was evaluated using a two-tailed student t-test, and a *P* value < 0.05 was considered significant. n=5. * is a shorthand symbol used to indicate the statistical significance level,* : p ≤ 0.05;** : p ≤ 0.01; ***: p ≤ 0.001; ***: p ≤ 0.0001.

### 3.2 Transcriptomic and gene expression analyzes

The percentage of bases with Q-Phred mass ≥ 20 and ≥ 30 were both above 97%. The GC content ranged from 48.76% to 56.85%, indicating good sequence quality and consistency (**Supplementary Table S1**).

Based on the sample clustering outlier detection analysis, all the samples are not outlier samples (**Supplementary Figure S1**). PCA analysis was used to elucidate disparities among wheat samples under heat stress (**Figure 2A**). The first two principal components, accounting for 54% and 30% of the variability, respectively, resulted in tight clustering within groups and dispersion between groups. The distribution of the group not subjected to heat stress (0 h) was significantly dispersed from that of the heat-stressed group, indicating significant differences in gene expression. Conversely, the distribution of groups subjected to heat stress for 0.5 h approximated that of groups subjected to heat stress for 3 h, while the distribution of groups subjected to heat stress for 24, 72, and 120 h approximated each other, indicating significant difference in gene expression between early (0.5–3 h) and late (24–120 h) heat-stressed groups. Based on |log2Fold Change| ≥ 1 and FDR < 0.05, we identified the DEGs between different materials at the same time point and within the same material across different time points. As the duration of heat stress increased, the number of DEGs also increased. We also conducted an intersection analysis on the differentially expressed genes between different materials at different heat stress time points. At the 3-hour, 24-hour, and 120-hour time points of heat stress, there were two common differentially expressed genes. At the 3-hour, 72-hour, and 120-hour time points, there was 1 common differentially expressed gene. At the 24-hour, 72-hour, and 120-hour time points, there was 1 common gene as well. (**Figure 2B**) After 0.5 hours of heat stress, compared with CK, ZNM1 had 1757 differentially expressed genes. Compared with CK, YM20 had 4674 differentially expressed genes. While compared with YM20, ZNM1 had 786 differentially expressed genes. After 3 hours of heat stress, there were 2678 differentially expressed genes between ZNM1 and CK, 1807 between YM20 and CK, and 891 between ZNM1 and YM20. After 24 hours of heat stress, there were 6929 differentially expressed genes between ZNM1 and CK, 6042 between YM20 and CK, and 1328 between ZNM1 and YM20. After 72 hours of heat stress, there were 8951 differentially expressed genes between ZNM1 and CK, 11224 between YM20 and CK, and 977 between ZNM1 and YM20. After 120 hours of heat stress, there were 9769 differentially expressed genes between ZNM1 and CK, 17761 between YM20 and CK, and 6924 between ZNM1 and YM20. As the heat stress time increased, the number of differentially expressed genes between different varieties and CK increased, while the number of differentially expressed genes between different varieties showed a trend of increasing first and then decreasing with the increase of heat stress time. (**Figure 2C**; **Supplementary Table S2**).

**Figure 2:**
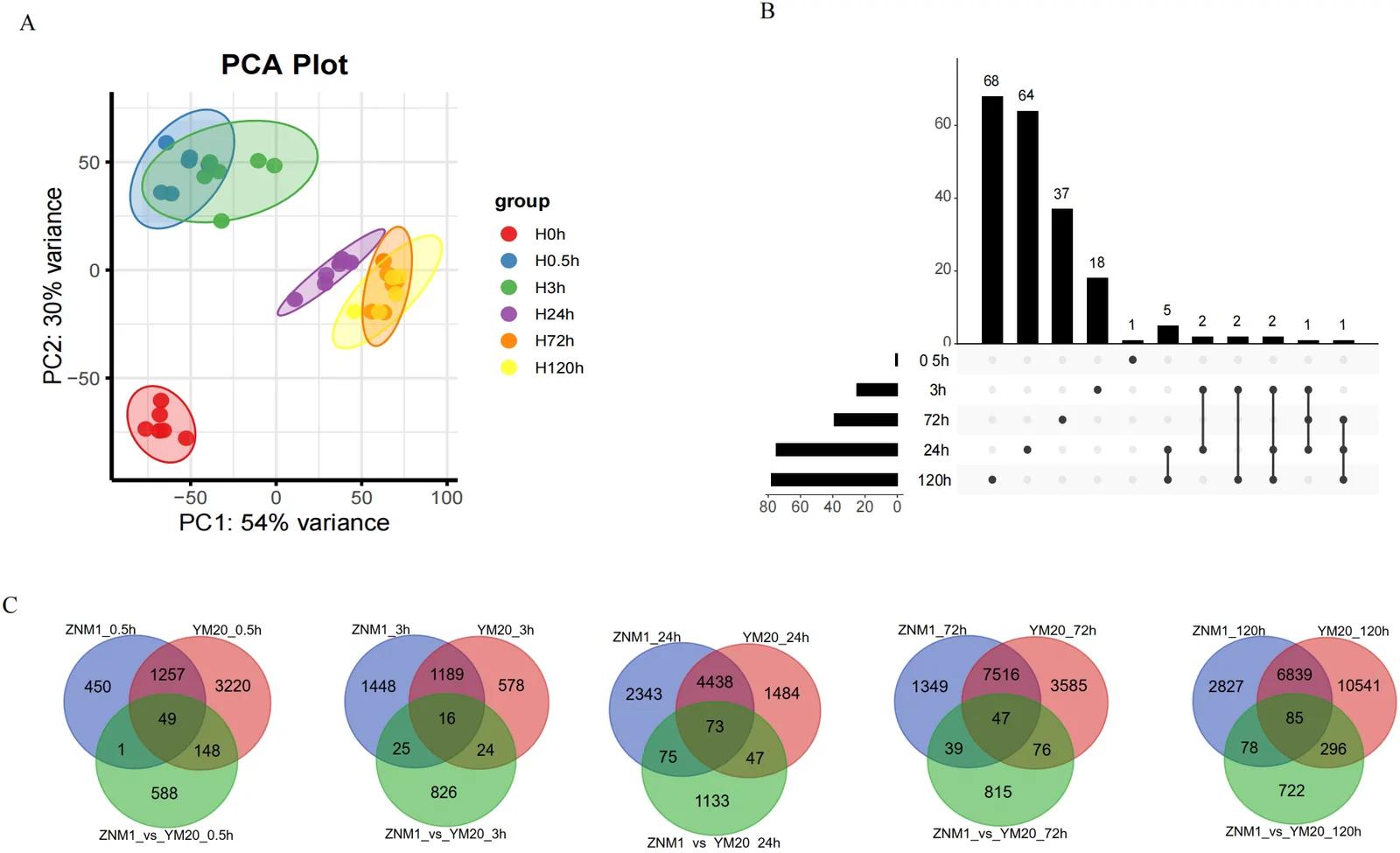
Differential gene analysis **(A)** Principal component analysis (PCA) of 36 leaf samples. PCA plots of leaf samples under heat stress (42 °C) at different treatment times (H0h, H0.5 h, H3h, H24h, H72h, and H120h). Triplicate samples were analyzed. **(B)** Diagram upset shows the intersection of differentially expressed genes at different time points after heat stress with those at other time points. Diagram. Left horizontal bar chart: The number of differentially expressed genes that were individually upregulated or downregulated at each time point. The below grid: represents the intersection situation of different time point combinations. Vertical bar chart above: The number of common differentially expressed genes in the corresponding combination. **(C)** It demonstrates the quantity of differentially expressed genes at each time point when different materials are compared with the material that has not undergone heat stress for 0 hours. Additionally, it shows the number of differentially expressed genes at the same time point when different materials are compared with one another. * is a shorthand symbol used to indicate the statistical significance level,* : p ≤ 0.05;** : p ≤ 0.01; ***: p ≤ 0.001; ***: p ≤ 0.0001.

### 3.3 Construction of co-expression network by WGCNA

After filtering non-differentially expressed genes, weighted gene co-expression networks were constructed for DEGs. Cluster analysis was performed with no outlier samples. The pickSoftThreshold function was used to calculate a suitable soft threshold for the weighting coefficient, meeting the criteria (*R*^2^ value close to 0.8 and ensuring gene connectivity). A soft threshold of 9 was selected for the co-expression network (**Figure 3A**). Dynamic cutting was employed to divide the network modules (**Figure 3B**). Highly similar small modules were merged, resulting in 20 modules. Modules were assigned different colors for differentiation. Among them, the red module contains the largest number of genes (13417), while the lightyellow module has the fewest genes (25).

**Figure 3:**
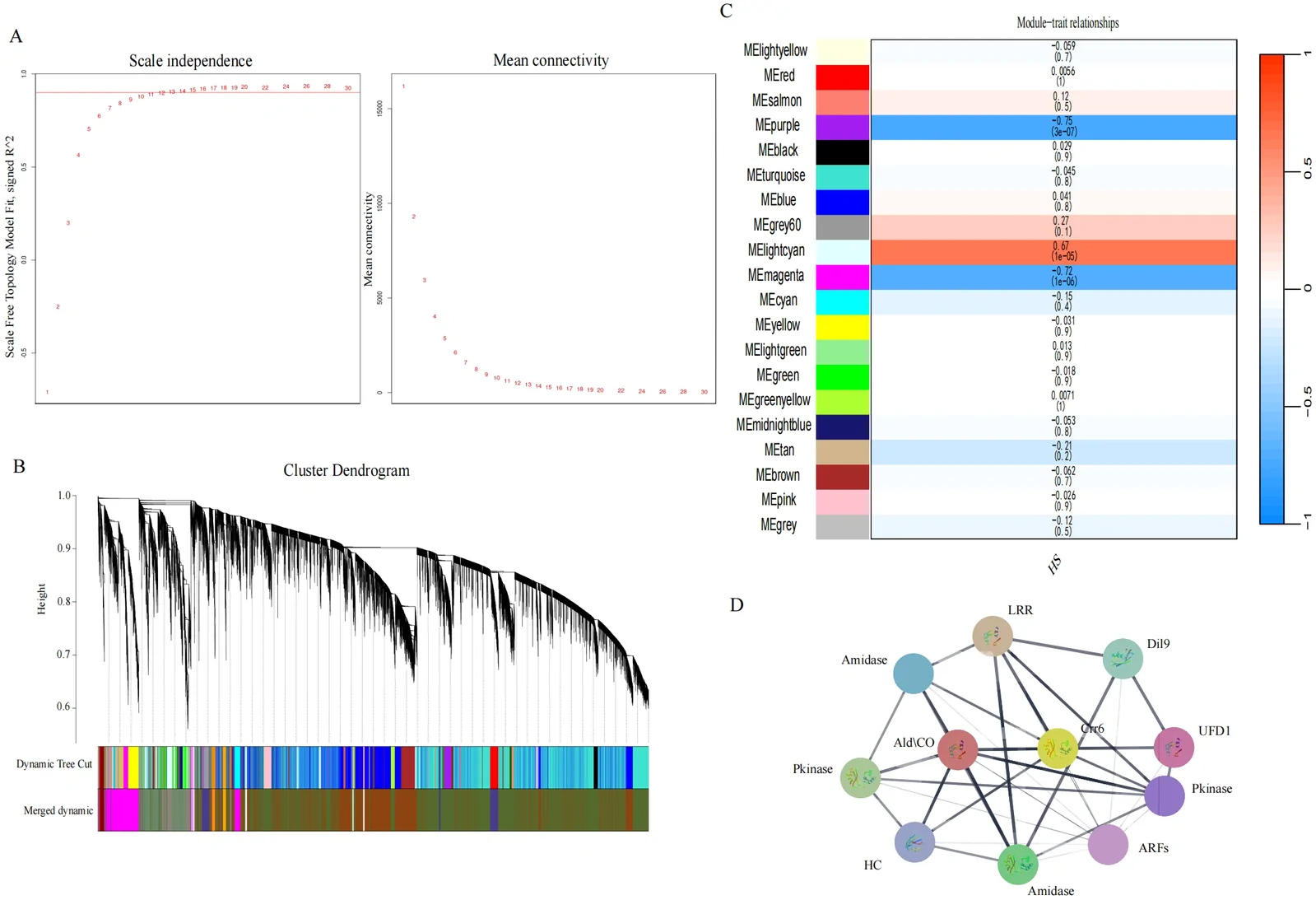
Weighted gene co-expression network analysis **(A)** Network topology analysis of wheat seedlings across different soft-thresholding powers. The left y-axis shows the scale-free topology model fit [R^2^ between log(k) and log(p(k)], and the right y-axis indicates the mean gene adjacency. A scale-free topology is attained at a soft-threshold power of 9. **(B)** Hierarchical clustering dendrogram and module identification. Dendrogram based on topological overlap matrix (TOM). Black branches represent gene clusters with similar expression patterns. Color bars indicate initial gene modules identified using the dynamic tree cut method, with each color representing a distinct module. The second color bar represents merged modules after combining highly similar initial modules. **(C)** Module-trait relationships. Heatmap shows correlation between WGCNA modules and heat stress (HS) in wheat. **(D)** Co-expression network of candidate genes.

### 3.4 Identification of specific modules associated with heat tolerance in wheat

Correlations between gene expression and treatment conditions were calculated, and three gene co-expression modules were identified (|*r*| > 0.50, *P* < 0.05). The lightcyan module had the highest positive correlation with heat tolerance traits (*r* = 0.67, *P* = 1e-05), while the purple and magenta modules had the highest negative correlations with heat tolerance traits (*r* = -0.75, *P* = 3e-07, *r* = -0.72, *P* = 1e-06). We screened the core genes in these three modules and analyzed their enrichment (**Figure 3C**). The genes in these three modules are considered to be related to heat resistance. We used the Cytoscape software to construct the regulatory network of these genes and found that only 11 genes might have regulatory interactions (**Figure 3D**).

### 3.5 Specific module enrichment analysis

To investigate functional and metabolic classifications in the heat stress response, we conducted GO and KEGG analyzes on genes in the lightcyan, purple, and magenta modules (**Figure 4**; **Supplementary Table S3**). We prioritized genes related to heat tolerance or stress resistance pathways. The lightcyan module is associated with drought and heat stress responses and ubiquitin fusion degrades protein homolog, including *TaDil19-1A, TaLRR-1A*, and *TaUFD1-6A*. The purple module is involved in NAD(P)H-related REDOX activities and inorganic anion transmembrane transport, including *TaPkinase-1B*, *TaPkinase-3B*, *and TaCrr6-6A*. The magenta module focuses on plant hormone signal transduction. All genes in these modules relate to stress tolerance, including *TaARFs-1D*, *TaAmidase.1-2D*, *TaAmidase.2-2D*, *TaHC-4A*, and *TaAld\CO-5B*. Based on enrichment analysis, we identified 11 candidate genes with significant expression differences between ZNM1 and YM20. We searched the IWGSC website for functional annotations and homologs in *Arabidopsis thaliana* or rice (*Oryza sativa*) for these 11 genes. Six have been homologs in rice or *Arabidopsis*, including *TaDil19-1A, TaLRR-1A, TaARFs-1D, TaPkinase-3B, TaAld\CO-5B, and TaCrr6-6A*, while the other five have not been characterized, including *TaUFD1-6A, TaAmidase.1-2D, TaAmidase.2-2D, TaPkinase-1B*, and *TaHC-4A.* These unreported genes are mainly related to ubiquitin fusion degradation protein homolog, auxin metabolic process, NAD(P)H-related REDOX activities, the expression levels of these five genes were significantly higher in ZNM1 than in YM20 after heat stress. We found that these genes respond to auxin signaling, NAD(P)H, and drought and heat tolerance-related pathways.

**Figure 4:**
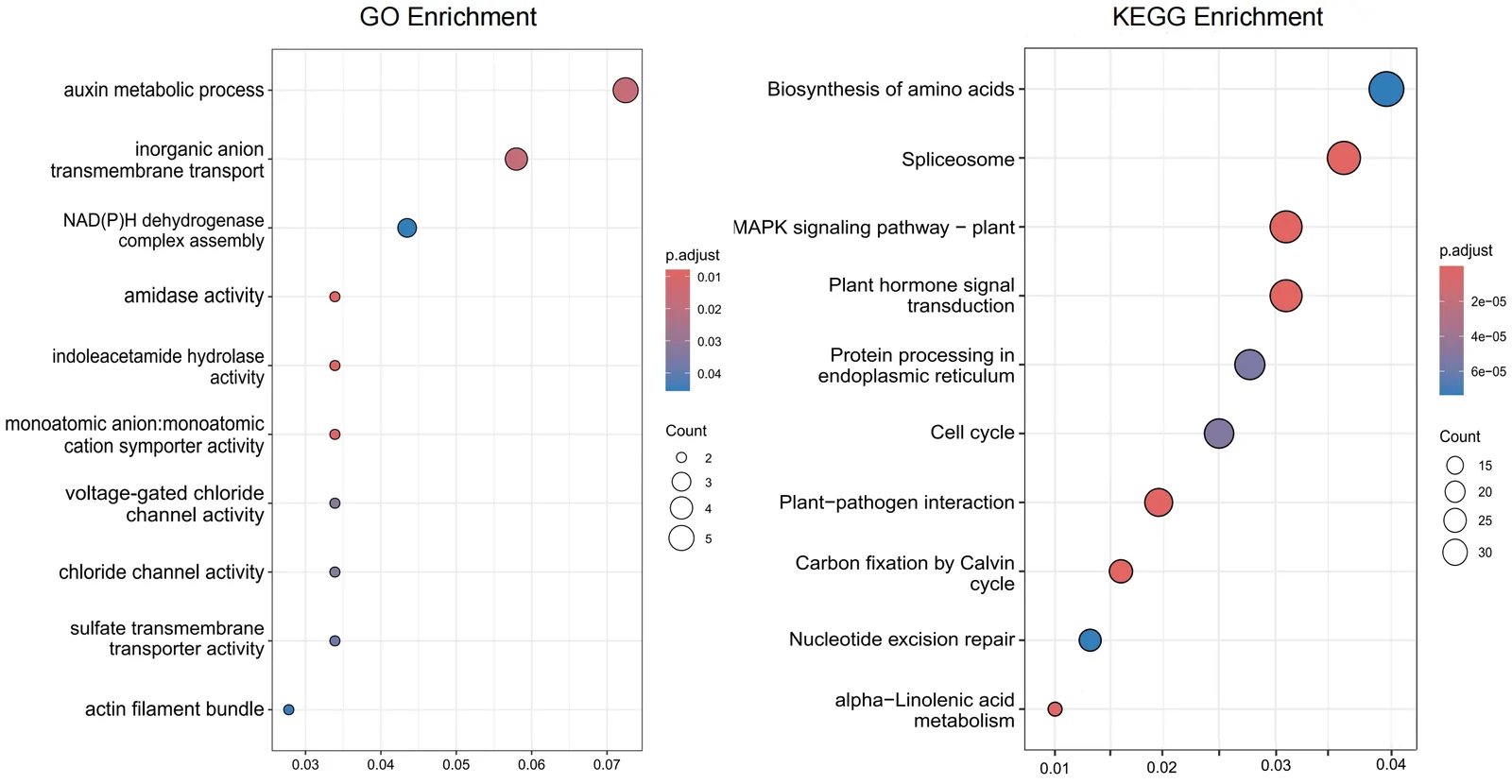
Functional analysis of relevant specificity modules. GO annotation of candidate genes in different modules.

### 3.6 RT-qPCR validation

RT-qPCR results indicated that 11 candidate genes at the seedling stage were differentially expressed in ZNM1 and YM20 (**Figure 5**). Among them, *TaDil9-1A*, *TaLRR-1A*, *TaUFD1-6A*, *TaPkinase-1B*, *TaPkinase-3B*, *TaAmidase.1-2D*, *TaAmidase.2-2D* and *TaHC-4A* were significantly highly expressed in ZNM1 compared to YM20 after heat stress, while *TaARFs-1D, TaAld\CO-5B*, and *TaCrr6-6A* were significantly highly expressed in YM20 compared to ZNM1 after heat stress. Furthermore, they also share a nearly identical trend, that is, from the initial 0.5 hours to 24 hours of heat stress, there is a significant difference in the expression of these genes between ZNM1 and YM20. However, as the duration of the heat stress increases, from 24 hours to 120 hours, the difference in the expression levels of these genes between ZNM1 and YM20 gradually decreases, and log2(Fold Change) values trending towards zero. Furthermore, the results of RT-qPCR were compared with the transcript abundance changes from RNA-seq data (**Supplementary Figure S2**).

**Figure 5:**
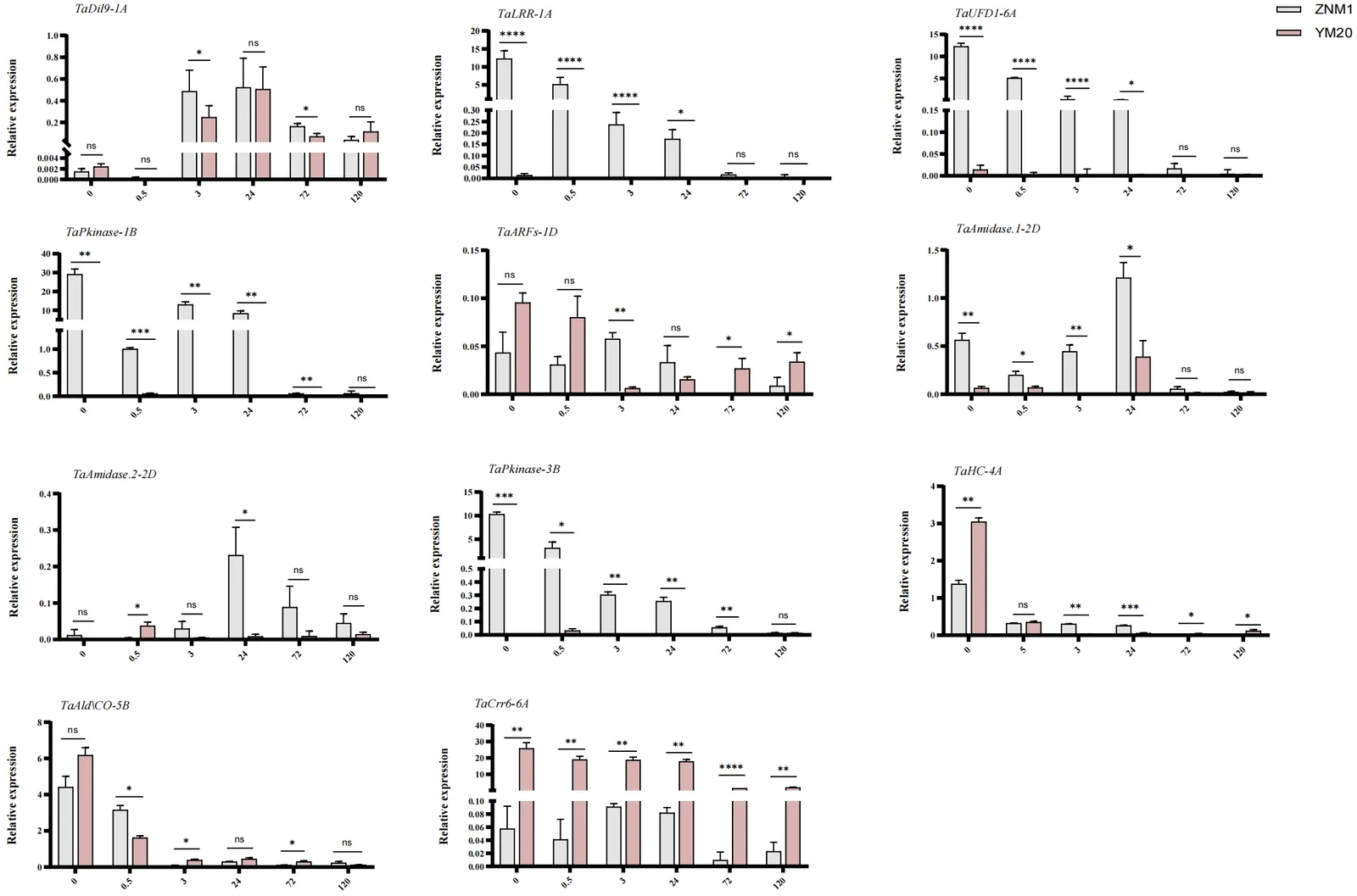
RT-qPCR verification of 11 candidate genes at the seedling stage. Genes: *TaDi119-1A*, Drought induced 19 protein, zinc-binding; *TaLRR-1A*, HEAT-like repeat; *TaUFD1-6A*, Ubiquitin fusion degrades protein 1 homolog; *TaPkinase-1B*, *TaPkinase-3B TaCrr6-6A*, NAD(P)H dehydrogenase complex assembly; *TaARFs-1D*, *TaAmidase.1-2D*, *TaAmidase. 2-2D*, *TaHC-4A*, *TaAld\CO-5B* auxin metabolic process. Gene expression levels were calculated using the 2^−ΔΔCt^ method, with wheat *β-TaActin* gene as the internal control. Data represents the mean ± SD from three independent replicates. Gene expression was normalized to internal control. n=3.

### 3.7 Expression verification of *TaARFs-1D* in yeast

Based on RT-qPCR results, four candidate genes—*TaDil9-1A*, *TaARFs-1D*, *TaAmidase.1-2D*, and *TaAmidase.2-2D*—were selected and subjected to heterologous expression in yeast to assess their heat resistance; however, only *TaARFs-1D* displayed a significant phenotype (**Supplementary Figure S3**). The *TaARFs-1D* sequences were ligated into the pGADT7 vector. After culturing the yeast cells at 28°C and inoculating them onto the culture medium, it could be observed that both the control empty pGADT7 and pGADT7-*TaARFs-1D* had complete colonies, and the number of colonies decreased as the gradient decreased. However, after inoculating them onto the culture medium after culturing at 42°C for 12 hours, it could be seen that the yeast of the empty pGADT7 was significantly less than that of the pGADT7-*TaARFs-1D*, suggesting that this gene has certain tolerance to heat stress (**Figure 6**).

**Figure 6:**
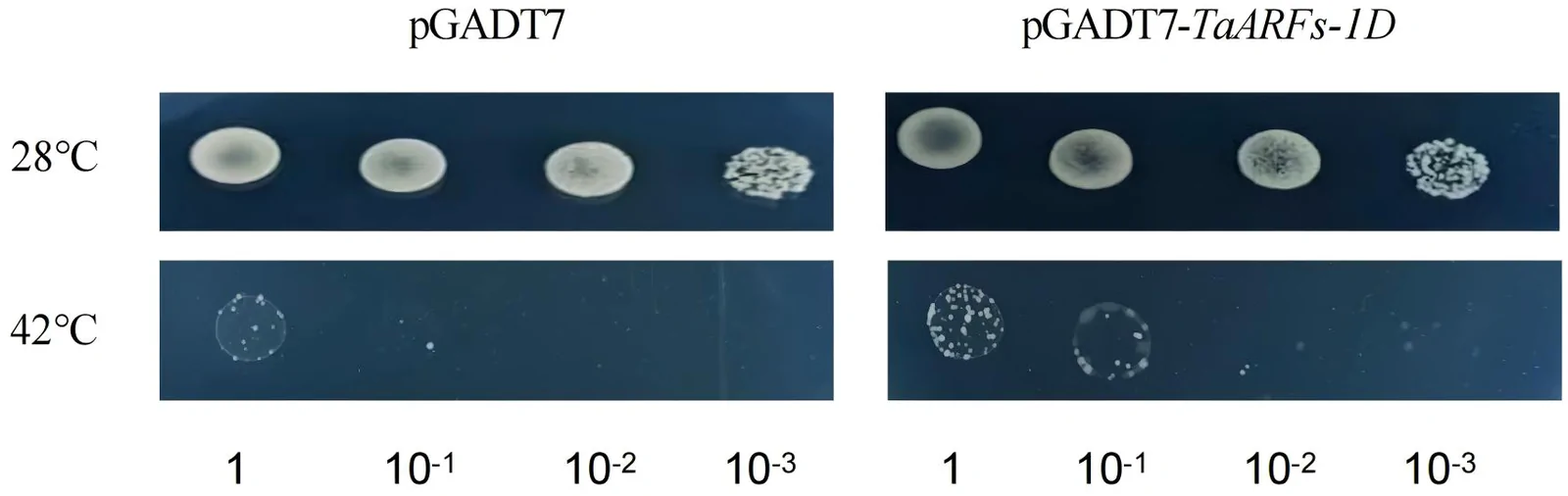
The growth tolerance of pGADT7 and pGADT7-*TaARFs-1D* in yeast after heat stress. n=3.

### 3.8 Expression dynamics of *TaARFs - 1D* genes underscore genotype and stress-specific adaptation

The haplotype network analysis indicated that *TaARFs* - *1D* possesses two primary haplotypes. In terms of geographical distribution frequency, Hap1 is more widespread among low-latitude varieties, whereas Hap2 is more prevalent among high-latitude varieties. This distribution pattern implies that this locus might have experienced environmental selection pressure associated with temperature. Although this is merely an observational outcome based on the existing resequencing data, it offers additional population genetics clues for *TaARFs* - *1D* as a candidate gene related to heat tolerance (**Figure 7**). Interestingly, *ARF* genes displayed distinct expression patterns in response to diverse developmental conditions (**Figure 8**). *TraesCS3A02G442000* and *TraesCS7A02G252000* demonstrated high expression, and *TraesCS1D02G506400LC*, *TraesCS2D02G577800*, and *TraesCS3D02G245400* displayed low transcript levels in distinct tissues, indicating their diverse functions in wheat development and growth. Half of the *TaARF* genes were highly expressed in response to heat stress in the HD2985 (thermotolerant) and HD2329 (thermosusceptible) wheat (**Figure 9**). Many *TaARF* genes were reduced after heat stress, which is similar with the RT-qPCR data. Furthermore, under the control and salt treatments, *TraesCS2D02G307600*, *TraesCS3D02G292100*, *TraesCS2B02G326400*, *TraesCS3A02G442000*, and *TraesCS3A02G449300* expression was higher in HD2329 than in HD2985 plants. Therefore, *TaARF* may be a key gene responsible for the difference in the response to heat stress between the two wheat genotypes.

**Figure 7:**
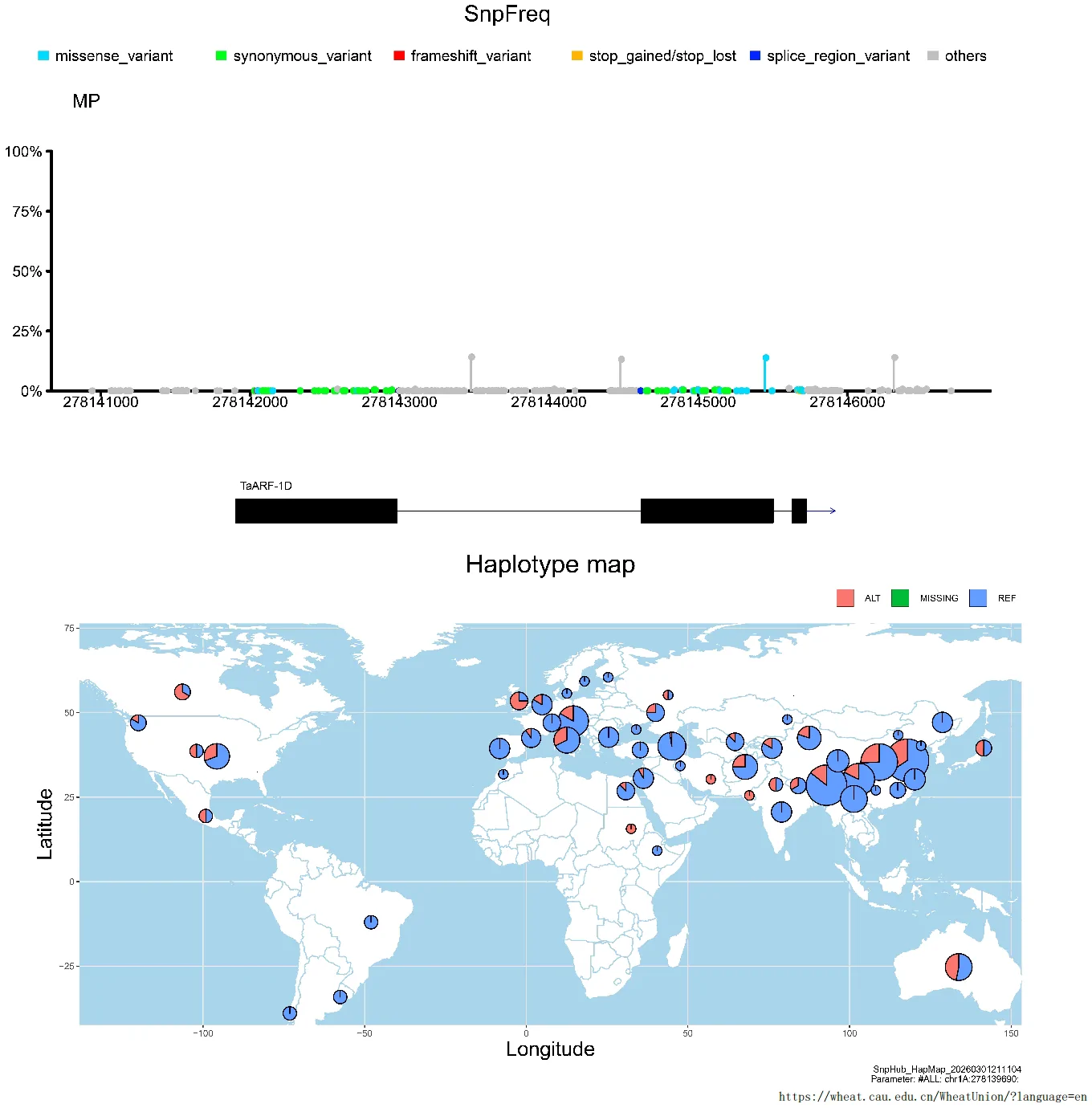
The variations at the TaARFs-1D locus in different varieties of wheat, as well as the global distribution of the two main haplotypes of TaARFs-1D, are shown. Blue represents the reference (REF) haplotype, and red represents the variant (ALT) haplotype.

**Figure 8:**
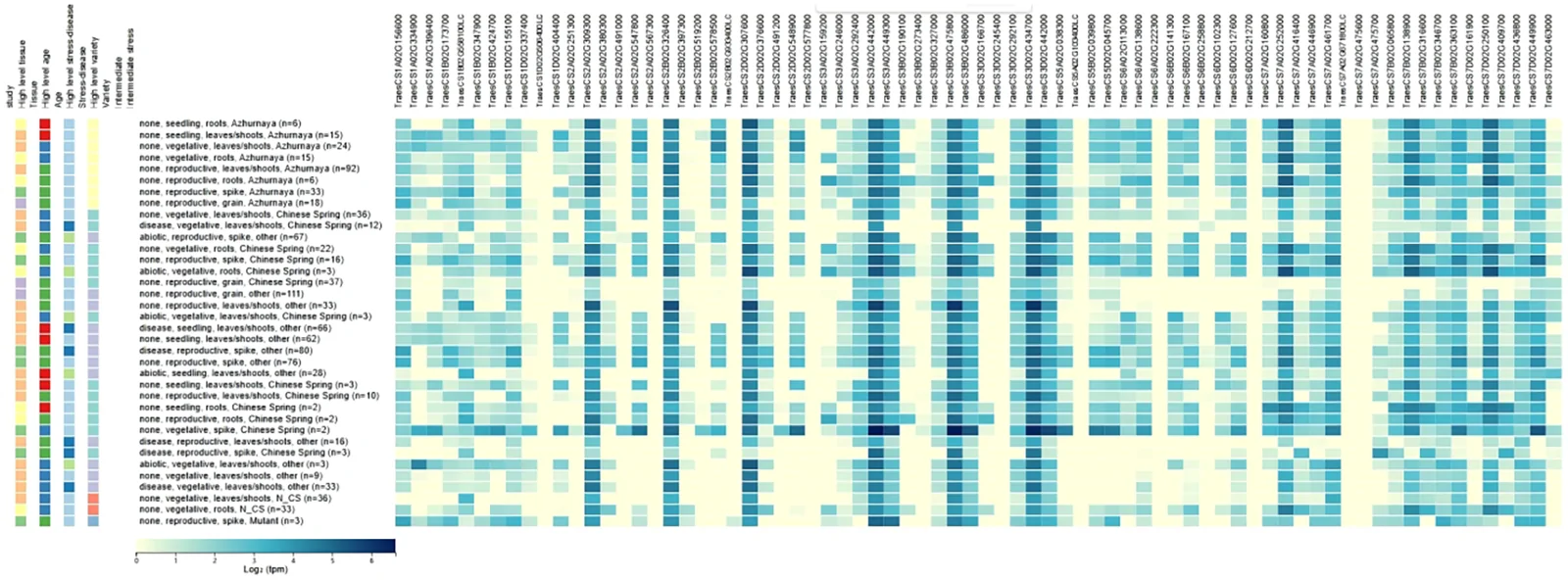
Expression of *TaARF* genes in various wheat tissues.

**Figure 9:**
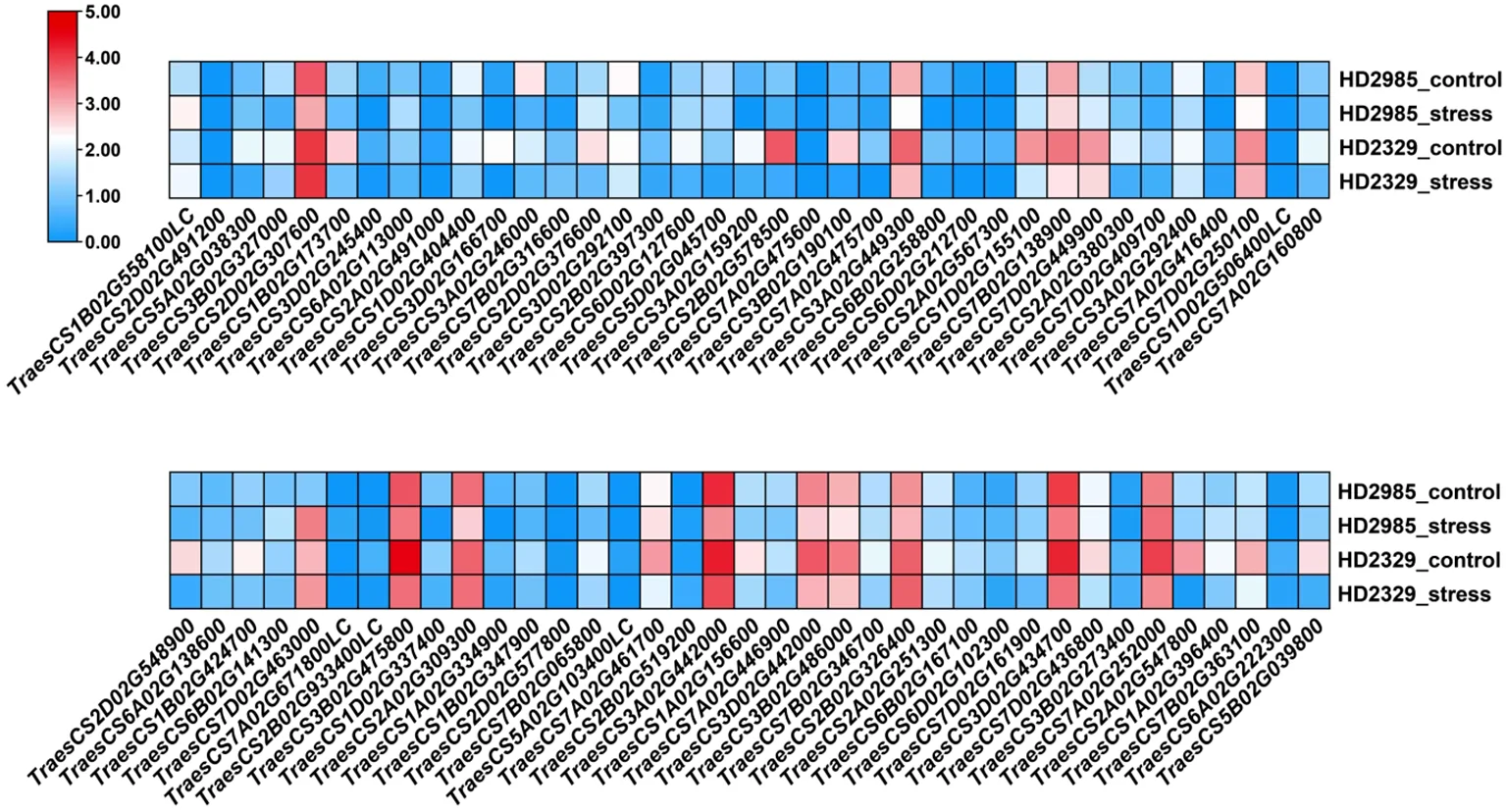
Expression of *TaARF* genes in HD2985 (thermo-tolerant) and HD2329 (thermo-susceptible) under heat stress.

## 4 Discussion

High temperature is a critical climatic factor that severely constrains plant growth, development, and productivity, leading to substantial yield losses in major crops, including wheat (Yadav et al., 2022; Abeed et al., 2022). In the middle and lower reaches of the Yangtze River, the intensification of global warming has resulted in frequent episodes of temperatures exceeding 30 °C during wheat flowering and grain filling, significantly threatening grain set and final yield. Despite extensive research on wheat thermotolerance, most studies have primarily focused on seedling-stage responses, with limited translation to breeding for field performance. Moreover, the relationship between seedling and reproductive-stage heat tolerance remains poorly understood. To bridge this gap, our study integrated phenotypic assessments across developmental stages with time-resolved transcriptomic analyses to identify and validate candidate genes underlying multi-stage heat tolerance.

Previous studies have shown that plant hormones, such as abscisic acid, auxin, ethylene, gibberellin, and brassinolide, are likely to be involved in the heat stress signal transduction processes of different species (Kotak et al., 2007; Larkindale and Knight, 2002; Larkindale et al., 2005; Alwutayd et al., 2024). With the development of sequencing technology, RNA-seq has been widely used in plant analysis (Kumar et al., 2020, 2015; Vishwakarma, 2016; Seni et al., 2021). In this study, GO (Lu et al., 2008) and KEGG (Du et al., 2016) were used to analyze DEGs enriched in auxin metabolism, NAD(P)H dehydrogenase complex, drought-responsive proteins, heat stress-responsive proteins, and ubiquitin fusion degradation proteins. Core genes were screened based on gene function annotations, with most core genes belonging to the *ARFs* (Gu et al., 2023), *Di119*, *Lrr* (Rathor et al., 2022), *UFD1*, *Pkinase* (Vij et al., 2008), *Crr6* (Yamori et al., 2011) gene families, which play important roles in heat stress. Since heat stress often accompanies drought stress in field conditions, we identified core genes associated with reactive oxygen species (ROS) scavenging in response to water deficit (Hamed et al., 2025; Abeed et al., 2023). For example, the ARF family has been shown to play a beneficial role in heat tolerance in ginger (Tong et al., 2025; Gao et al., 2024). However, the specific function of TaARF in wheat, particularly its role across different developmental stages, has not been well characterized. Our study provides evidence that TaARF is a hub gene in a co-expression module associated with auxin metabolism, and its heterologous expression in yeast conferred heat tolerance. This is consistent with findings in Arabidopsis, where ARF mutants exhibit altered stress responses, but it extends the knowledge by suggesting a conserved role for ARF in thermotolerance across species and developmental stages in wheat. Additionally. NDH-dependent cyclic electron flow in Crr6-deficient rice plays a crucial role in photosynthesis and growth under low-temperature conditions. NAD(P)H assembly genes show stronger co-expression with yield stability traits, implicating energy metabolism as an underestimated component of lifelong heat adaptation (Fedotova et al., 2023). This suggests that genes responding to other stresses are upregulated under heat stress.

Beyond the functional characterization of individual genes, the integration of time-resolved transcriptomics with WGCNA in this study provides broader methodological and biological insights into wheat heat tolerance. Compared with conventional DEG-based analyses, WGCNA effectively condenses thousands of differentially expressed genes into biologically meaningful co-expression modules, reducing false-positive rates and uncovering non-canonical pathways that are often overlooked in routine transcriptomic screenings (Zhang et al., 2025). Unlike most studies that focus on a single developmental stage, our multi-time-point design (0.5–120 h) captured the temporal dynamics of the heat stress response, revealing that the transcriptional divergence between ZNM1 and YM20 was most pronounced during the early phase (0.5–24 h) and gradually converged thereafter. This pattern suggests that the heat-tolerant genotype possesses a more rapid and sustained protective transcriptional activation. At the gene level, the identification of TaARF as a hub gene is consistent with findings in rice, where OsARF genes were implicated in cross-tolerance to heat and drought.

Collectively, our findings demonstrate that sustained thermotolerance in wheat is mediated by developmentally persistent regulation of auxin signaling homeostasis, NAD(P)H dehydrogenase complex assembly, and antioxidant defense systems. Through comparative transcriptomics of ZNM1 and YM20 at the seedling stage, we identified 11 core candidate genes with conserved stress-responsive expression patterns, including ARF transcription factors implicated in auxin-mediated grain development and Crr6-like factors involved in mitochondrial NAD(P)H dehydrogenase assembly. Functional enrichment analyses corroborated ZNM1’s superior yield stability under heat stress, with 90.05% TGW retention compared to a 15.47% loss in YM20.

RT-qPCR validation confirmed that most seedling-stage candidate genes exhibited expression patterns consistent with RNA-seq TPM values, reinforcing the robustness of our transcriptomic analyses. These genes represent promising targets for molecular breeding or genome editing aimed at enhancing wheat thermotolerance throughout its life cycle. It is noteworthy that, at certain time points, the induced expression level of *TaARFs-1D* in the heat-sensitive cultivar YM20 was even higher than that in the heat-tolerant cultivar ZNM1. This pattern does not necessarily contradict the proposed positive role of *TaARF* in heat tolerance. Rather, it may reflect the complex relationship between the magnitude of stress response and the ultimate tolerance phenotype. In YM20, the higher degree of gene induction might represent a “compensatory” or “stress-sensing” response, indicating that its system is in a more intensely perceived stress state (Chen et al., 2026). However, due to potentially less efficient downstream signal transduction or protective mechanisms, this strong transcriptional response may fail to effectively translate into heat tolerance. In contrast, in ZNM1, although the induction level of this gene is relatively lower, its genetic background or coordinated regulatory network may enable effective protective functions to be executed at a lower expression threshold.

It is noteworthy that among the 11 candidate genes selected, the haplotype analysis based on the public database shows that *TaARF* is the sole gene that demonstrates non-synonymous SNP variations between the heat-resistant variety ZNM1 and the heat-sensitive variety YM20. Genetic structural variations typically signify functional differentiation. Given that it belongs to the key stress signal family of auxin response factors, we prioritized an initial exploration of its heterologous expression. Although yeast experiments indicated its potential for heat resistance, we recognize that other highly expressed genes (such as *TaLRR*) also hold significant research value, and the specific functional verification of these genes will be the focus of our subsequent transgenic work.

## Data Availability

The raw sequence data reported in this paper have been deposited in the Genome Sequence Archive (Genomics, Proteomics & Bioinformatics 2025) in National Genomics Data Center (Nucleic Acids Res 2025), China National Center for Bioinformation / Beijing Institute of Genomics, Chinese Academy of Sciences (GSA: CRA050902) that are publicly accessible at https://ngdc.cncb.ac.cn/gsa.
